# Graph convolutional networks for inferring cell-cell communication from spatial transcriptomics data

**DOI:** 10.1093/bioadv/vbag101

**Published:** 2026-04-09

**Authors:** Roman Kouznetsov, Jackson Loper, Jeffrey Regier

**Affiliations:** Department of Statistics, University of Michigan, Ann Arbor, MI 48109, USA; Department of Statistics, University of Michigan, Ann Arbor, MI 48109, USA; Department of Statistics, University of Michigan, Ann Arbor, MI 48109, USA

## Abstract

**Motivation:**

Single-cell spatial transcriptomics provides gene expression measurements of individual cells while preserving their spatial positions within tissue. Cell-cell communication (CCC) can be inferred by comparing the predictions of held-out gene expression levels by a pair of models: one that incorporates cellular neighborhood information and another that does not. The performance gap indicates the influence of CCC. However, existing methods that adopt this general approach often rely on spatially informed models that use simplistic representations of spatial context. This reliance on such representations does not merely lead to suboptimal predictions: it undermines the validity of the model comparison itself, which hinges on the accurate estimation of conditional expectations.

**Results:**

We propose using a graph convolutional network (GCN) as a highly expressive spatially informed model, with cells as nodes and spatial proximity as edges. In semi-synthetic datasets, we show that several existing approaches relying on simplistic neighborhood features can produce spurious inferences about CCC, whereas our GCN-based approach avoids these pitfalls. In MERFISH and Xenium mouse brain tissue, our method identifies genes with known spatial variation, suggesting that it successfully infers CCC-affected genes.

**Availability and implementation:**

Code to reproduce our results is available from https://github.com/prob-ml/spice.

## 1 Introduction

Single-cell spatial transcriptomics is a revolutionary technology that measures both the gene expression and the positions of individual cells within a tissue sample (Marx 2021). The availability of these cell positions allows distances between cells to be computed and used to model cell-cell communication (CCC). Ligands emitted by sender cells can trigger a variety of cell responses in receiver cells. Prior to single-cell spatial transcriptomics, gene expression was commonly measured using bulk RNA-seq (Mortazavi *et al*. 2008, Wang *et al*. 2009) and single-cell RNA-seq (Tang *et al*. 2009), neither of which preserves cell positions. Before the availability of this spatial information, modeling CCC effects involved constructing networks using prior biological knowledge, such as known ligand-receptor interactions or pathway databases (Fortelny and Bock 2020).

While ligand-receptor interactions and signaling mechanisms in CCC are well studied, quantifying these effects in individual tissues remains an open challenge and has important applications (Foster *et al*. 2021). For example, knowing the extent of spatial dependence among genes is useful for identifying genes that drugs can target to inhibit or activate specific pathways, thereby altering disease progression (Yang *et al*. 2024). Statistical frameworks for ranking genes based on improvement in predictive performance from including CCC information may streamline drug development by prioritizing high-potential targets and reducing experimental validation costs. Even non-target genes with spatially dysregulated expression in diseased tissue may help guide experiments. For example, if genes known to drive cancer display clear spatial patterns, it may suggest that the tumor microenvironment modulates gene expression and influences tumor progression (Tirosh *et al*. 2016). Understanding the spatial dependence of gene expressions is essential to understanding cellular heterogeneity within tissues (Satija *et al*. 2015).

In this work, we examine cell-cell communication (CCC) through the lens of model selection. We consider gene expression at the single-cell level and distinguish two drivers of expression: local expression, defined as the expression level of each gene within a specific cell, and neighborhood expression, defined as the gene expression levels observed in neighboring cells. To assess the impact of spatial context on gene expression, we compare two types of models: spatially informed models, which explicitly incorporate the influence of neighboring cells on local gene expression, and spatially ignorant models, which assume that local gene expression is independent of neighboring cells. The comparison between these two classes of models is based on their predictive performance. For each response gene of interest, we attempt to predict its expression using both model types. Genes involved in CCC are identified as those for which the spatially informed model yields substantially more accurate predictions than the spatially ignorant model, indicating that intercellular interactions within their spatial environment influence their expression.

**Figure 1:**
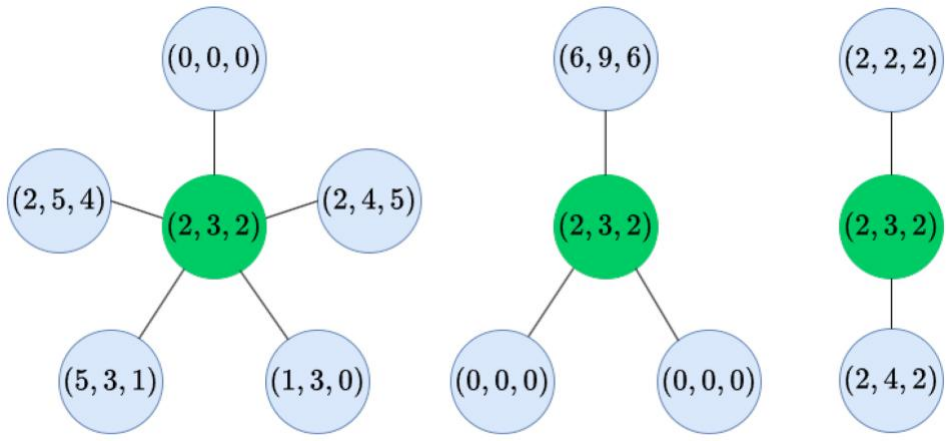
Illustration of the limitations of a manually engineered, fixed-dimensional representation of neighboring gene expression. Each circle represents a cell, with target cells (green, in the center) receiving signals from neighboring cells (blue). The neighborhood representation for each target cell is produced by averaging the gene expression vectors from neighboring cells. This representation is the same for every target cell, even though their neighborhoods differ greatly in many ways.

The effectiveness of this model-selection approach for identifying spatially regulated genes depends on the quality of the models being used. A misspecified spatially informed model may fail to produce better predictions than a spatially ignorant model, even in the presence of CCC-based dependencies. In this case, the model-selection approach may fail to identify that genes are regulated through CCC. The model-selection approach, therefore, depends on high-quality spatially informed models. Models relying on manually engineered neighborhood summaries discard relevant spatial information, which contributes to model misspecification (Fig. 1).

We propose to use graph convolutional networks (GCNs), which are highly flexible, as the spatially informed model class. Unlike several prior CCC inference methods that rely on fixed or manually engineered neighborhood summaries, GCNs operate directly on the spatial graph, preserving the full structure of local neighborhoods. We demonstrate that GCNs can effectively leverage spatial context to predict response gene expression, making them more useful for drawing robust conclusions about spatial dependence and CCC. Furthermore, the added flexibility of GCNs ensures that both the spatially ignorant and spatially informed models are sufficiently expressive, so that differences in predictive performance can be attributed to the inclusion of spatial information rather than to representational constraints. GCNs use similar inputs to preceding work but allow the model to learn which aggregations of neighboring expressions are useful for downstream predictions, rather than committing to a predetermined summary statistic.

### 1.1 Related work

Since the emergence of single-cell spatial transcriptomics in 2015, many methods have been proposed to gain insights into CCC from this type of data. We summarize established model selection approaches for isolating spatial effects (Section 1.1.1) and discuss how GCNs have already been used in spatial transcriptomics (Section 1.1.2).

#### 1.1.1 Models for isolating spatial effects

A growing body of work seeks to quantify CCC by comparing spatially informed models to spatially naive baselines or by disentangling spatial variance from total variance.

COMMunication analysis by Optimal Transport (COMMOT) (Cang *et al*. 2023) infers cell–cell communication (CCC) in spatial transcriptomics by solving an optimal transport problem that incorporates spatial distance and ligand/receptor expression while modeling competition among molecular signals. To assess CCC effects, a random forest predicts target gene expression using non-target genes and a CCC vector summarizing local communication. Gini importance is used to infer signaling impact, assuming both that the CCC vector captures relevant spatial context and that feature importance reflects predictive relevance—assumptions that may not hold in practice (Strobl *et al*. 2007).

nnSVG (Weber *et al*. 2023) identifies genes that vary continuously across tissue samples in a regression setting, using a Gaussian process to encode spatial correlation through a covariance structure that decays with distance and assesses spatial influence by conducting a likelihood ratio test between a null Gaussian process (with no intercellular correlation) and a Gaussian process with a spatially informed correlation structure. The expressiveness of Gaussian processes is limited by the use of a fixed class of kernel functions that define spatial similarity. In contrast, GCNs learn how to weight neighboring signals through message passing, rather than relying on a predefined formulation of similarity.

Spatial Interaction Modeling using Variational Inference (SIMVI) (Dong *et al*. 2025) uses variational inference to approximate posterior distributions over latent intrinsic and spatial variation for each cell. After performing archetypal analysis to transform spatial variation into archetype weights, the archetype weights and log-transformed expression values are regressed against intrinsic variation. The $R^{2}$ between the predicted expression and the true expression represents the variance explained by intrinsic variation alone. To obtain the spatial effect, the residuals of the expressions are regressed against the residual archetype weights as well as an interaction between archetype weights and intrinsic variation. The $R^{2}$ of this regression represents the result for spatial variation. While the approximate posteriors have sufficient flexibility, SIMVI assumes a specific parametric form for intrinsic and spatial variation. Furthermore, the spatial effect is derived by estimating latent variables from a single trained generative model rather than comparing separately trained models that explicitly isolate spatial effects, which could be problematic if variability is attributed to spatial effects when it instead arises from intrinsic variation that the generative model does not capture.

SPAtial CEllular NETworks from omics data (SpaCeNet) (Schrod *et al*. 2024) model intercellular gene dependencies using a Gaussian graphical model in which a precision matrix is parameterized with the assumption that intercellular associations are related via some function of the Euclidean distance between cells. Their precision matrix is broken down into intracellular and intercellular components: $\Lambda =\Lambda_{\text{within}\;}+\Lambda_{\text{between}}.$ Comparing results with estimated parameters for $\Lambda_{\text{between}\;}$ to those generated by setting $\Lambda_{\text{between}}=0$ quantifies the CCC effect under the SpaCeNet model. However, the reliance on a structured precision matrix imposes strong assumptions about the form of gene–gene interactions, and a predefined function for cell-cell relationships as a function of Euclidean distance may limit flexibility when modeling complex, nonlinear signaling relationships.

Mixture of Experts for Spatial Signaling genes Identification (MESSI) (Li *et al*. 2021) leverages a mixture-of-experts model for gene prediction. Like our proposed approach, MESSI adopts a model-selection perspective, comparing spatially informed predictions with spatially ignorant predictions to identify signaling genes responsible for response expressions by evaluating the learned coefficients from each expert. However, in contrast to our proposed approach, MESSI represents neighborhood expression by aggregating it into summary statistic representations that may fail to capture the full complexity of spatial organization. As shown in Fig. 1, such summaries can produce identical neighborhood representations from different spatial structures.

#### 1.1.2 GCNs for spatial transcriptomics

Graph convolutional networks have been used in various ways to gain insights into CCC from spatial transcriptomics data.

Graph Convolutional Neural networks for Genes (GCNG) (Yuan and Bar-Joseph 2020) casts detecting CCC gene relationships as a GCN link prediction problem. In contrast, our approach defines edges and edge attributes based on cell proximity and uses them to predict gene expression.

SpaGCN (Hu *et al*. 2021) uses a GCN to learn an embedding that is used for spatial domain clustering. GraphST (Long *et al*. 2023) trains a graph neural network with self-supervised contrastive learning, enhancing its ability to generate spatially informed representations for both clustering analysis and cell-type deconvolution. In contrast, we use GCNs to predict response expressions conditioned on neighboring ligand and receptor activities, which is a regression problem.

Node-centric expression models (NCEM) (Fischer *et al*. 2023) represent spatial relationships between cells or spots using proximity graphs to model how neighborhood composition influences gene expression. The NCEM variant most similar to ours in terms of its predictor–target formulation, called Nonlinear Ligand-Receptor NCEM, uses ligand and receptor expression as input but performs worse than a spatially ignorant baseline on a dataset with known CCC activity, in a comparison that is difficult to interpret due to the lack of model nesting. In contrast, our proposed method uses a graph convolutional network (GCN) to directly compare spatial and non-spatial models, enabling the systematic identification of individual genes whose expression levels are driven by CCC.

### 1.2 Our contribution

We develop a method called SPatial Inference of Communication Effects (SPICE) for identifying spatially regulated genes. SPICE uses a GCN to predict gene expression in spatial transcriptomic data. By explicitly comparing spatially ignorant and spatially informed GCN regression models, SPICE quantifies the predictive value of spatial context to identify CCC-affected genes.

## 2 Materials and methods

### 2.1 Datasets

**Figure 2:**
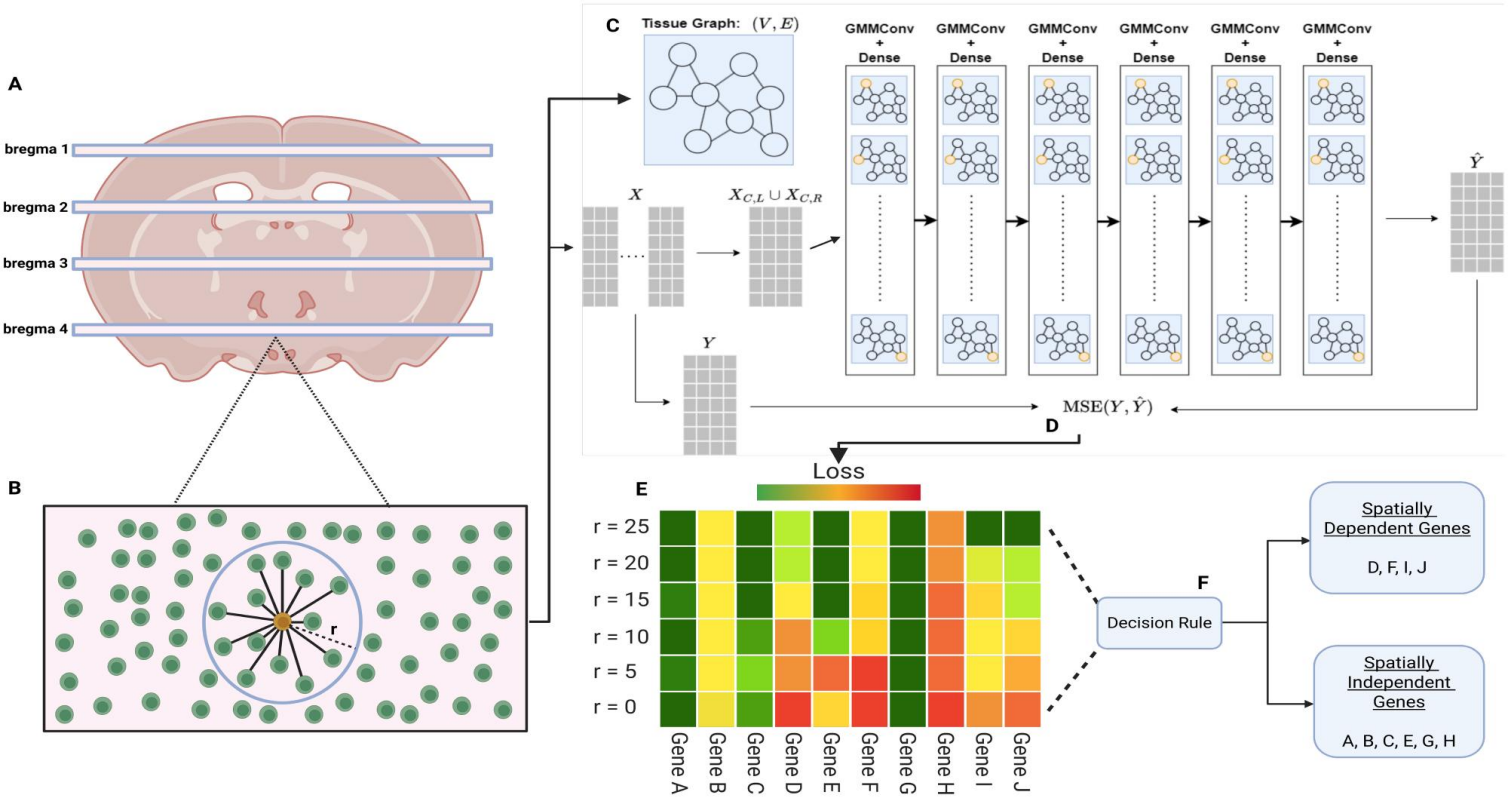
The SPICE method. (A) Tissue extraction. Source tissues are collected from the data source at varying locations. (B) Graph creation. For each target cell *i* (orange) measured in the tissue graphs, a neighborhood is created by adding bidirectional edges from *i* to all cells less than *r* µm away. (C) Forward model. Each graph is input to the model, with ligands and receptors as predictors. After several convolution and dense layers, the model outputs predictions for the response genes. (D) Model evaluation. The observed response expressions for all genes across all cells (*Y*) are compared with SPICE’s predictions ($\hat{Y}$) using MSE. (E) Spatial analysis. SPICE model evaluation for multiple neighborhood radii. Downstream analysis using this information can help identify spatially variable genes affected by CCCs. (F) Identifying spatially dependent genes. By comparing model performances from a spatially ignorant model (r=0 µm) and a spatially informed model (r=25 µm depicted here), we can identify or rank genes with spatial dependence.

We assess SPICE’s performance on two large-scale spatial transcriptomics datasets. The first is the MERFISH hypothalamus dataset (Moffitt *et al*. 2018). This dataset provides high-resolution single-cell spatial data, including approximately 1 million cells profiled across the hypothalamic preoptic region of 36 different mice, yielding 181 unique tissues. These cells are collected across several tissue slices across animals and across different locations within an animal (Fig.  2A). For each cell, the dataset contains its (i) slice, (ii) position, (iii) cell type, and (iv) gene expression for 161 genes (71 ligands and receptors; 84 responses; and 6 controls).

The second dataset we analyze is the Fresh Frozen Mouse Brain Replicates dataset provided by the Xenium platform (10x Genomics 2023). In contrast to earlier spatial transcriptomics platforms, Xenium data has both high spatial resolution and measurements of more genes. The Fresh Frozen Mouse Brain dataset contains 248 relevant genes, 100 of which are ligands and receptors.

Whereas MERFISH typically images tissue slices tens of microns apart, Xenium captures multiple adjacent sections with continuous spatial coordinates across all three dimensions. This third spatial dimension allows us to understand SPICE’s performance in settings with additional non-planar directional information. Signals from neighboring cells can come from any direction, so we assess SPICE’s ability to consider all plausible cell-cell communication (CCC) interactions. This Xenium dataset does not include ligand and receptor annotations, so to classify genes as ligands, receptors, or response genes, we query each gene in each of the following databases to determine if a gene is a known ligand or receptor: OmniPath Intercellular Roles and OmniPath Ligand-Receptor Interaction Database (Türei *et al*. 2021), ConnectomeDB2020 (Hou *et al*. 2020), and the Jin 2021 Mouse Ligand-Receptor Compendium (Jin *et al*. 2021). These curated sources provide broad and complementary coverage of ligand and receptor annotations.

For all datasets considered, the genes are annotated by type: ligand, receptor, or response. Ligands and receptors are treated as inputs and are preprocessed with log1p transformations. Response genes are left untransformed and used as prediction targets.

### 2.2 Graph creation

To apply GCNs to single-cell spatial transcriptomics data, we construct graphs in which cells are nodes and edges are defined by cells’ spatial proximity. Specifically, we include an edge in the graph if the distance between any two cells (*i*, *j*) is below a threshold *r*. Each edge is a potential channel for CCC. Each node (cell) is associated with the following node attributes: expression levels of predictor genes (ligands and receptors), response gene expression levels, and, optionally, cell type annotations.

### 2.3 GCN architecture

Graph convolutional networks (GCNs) are a type of neural network that operates on graph-structured inputs rather than tabular or grid-like inputs, mapping node attributes to per-node predictions (Chami *et al*. 2022). Unlike traditional neural networks and convolutional neural networks, GCNs are spatially informed and typically rotation invariant (Mac and Nguyen 2021). By stacking multiple layers, GCNs integrate information from nodes progressively further away, capturing both immediate neighborhood interactions and complex, long-range relationships that reflect signal propagation through biological pathways.

We propose a GCN architecture for use in a spatial transcriptomics setting. The proposed architecture is defined using an operator developed by Monti *et al*. (2017), known as the Gaussian mixture model convolutional operator (GMMConv). The GMMConv operator is defined in terms of many simpler units, referred to as “kernels.” Each kernel *k* is characterized by learnable parameters: a matrix $\Theta_{k}\in R^{d_{\ell +1}\times d_{\ell }}$, a mean vector $\mu_{k}\in R^{\dim(e_{i,j})}$, and a covariance $\Sigma_{k}\in R^{\dim(e_{i,j})\times \dim(e_{i,j})}$. The GMMConv operator uses the kernel parameters, together with edge attributes e and node attributes x, to produce transformed node attributes $x^{\prime }$. Specifically, for node *i*, let N(i) denote the set of its neighboring nodes. Then, the GMMConv operator computes


$$x_{i}^{\prime }=\frac{1}{\left|N(i)\right|}\sum_{j\in N(i)}\frac{1}{K}\sum_{k=1}^{K}w_{k}\left(e_{i,j}\right)⊙\Theta_{k}x_{j}=:\text{GMMConv}(x_{i}),$$

where each $w_{k}$ is a weighting function defined by


$$w_{k}(e)=\text{exp}\;\left(-\frac{1}{2}{\left(e-\mu_{k}\right)}^{⊤}\Sigma_{k}^{-1}\left(e-\mu_{k}\right)\right).$$

The architecture of the GCN in SPICE contains three hidden layers, each constructed as the sum of a GMMConv and a fully connected layer that takes in the output (transformed node attributes) from the previous layer, followed by a ReLU activation:


$$H^{\left(\ell +1\right)}=\mathrm{ReLU}(\mathrm{GMMConv}(H^{\left(\ell \right)})+H^{\left(\ell \right)}W^{\left(\ell \right)}),$$

where $H^{\left(\ell \right)}\in R^{|V|\times d_{\ell }}$ denotes the node feature matrix at layer ℓ. The final layer represents the predicted response gene expressions. The predictions are evaluated by computing the MSE between the model outputs and the true response gene expressions. We use polar coordinate vectors describing the distance between pairs of cells as the edge attributes


$$e_{i,j}=\left(\sqrt{{\left(x_{j}-x_{i}\right)}^{2}+{\left(y_{j}-y_{i}\right)}^{2}},\arctan\left(\frac{y_{j}-y_{i}}{x_{j}-x_{i}}\right)\right)$$

and use K=10 kernels for each GMMConv layer. The GCN is trained using the Adam optimizer with a learning rate of 0.001 and is early-stopped after 10 training epochs if there is no improvement on the validation set. The supplementary material (Section 1) provides additional details about the model architecture, hyperparameter tuning, and memory usage.

The added flexibility of allowing each pair of connected nodes in a graph to have a weight learned by a network has been shown to outperform other convolutional approaches to applied deep learning problems, including classical Euclidean convolutional neural networks (CNNs), spectral CNNs, GCNs, and diffusion CNNs (DCNN) (Monti *et al*. 2017). Furthermore, several commonly used message-passing operators can be represented as a GMMConv operation with careful edge-attribute selection.

### 2.4 Identifying spatially regulated genes

To identify CCC-affected genes, first create graphs for each slice of the spatial transcriptomics data for each of a variety of neighborhood radii. For each radius, split the resulting collection of graphs into training, validation, and test sets. Then, for each radius, use the training graphs to train a GCN (cf. Section 2.3) to estimate the expected response gene expressions conditioned on the other node attributes: ligand expression, receptor expression, and cell type annotations. Use the validation graphs to tune the GCN hyperparameters. Finally, evaluate the mean squared error (MSE) performance of the trained GCN on the test graphs, yielding an MSE value for each response gene and each radius, *r*. Comparing these values yields insight into which genes’ expression depends on the behavior of nearby cells; if a response gene’s expression depends on neighborhood expression, we expect lower MSE values for predictions from graphs with larger *r*. This method is illustrated in Fig. 2.

This approach can be understood formally as performing model selection. Let $Y_{c,g}$ denote the expression of a response gene *g* for a target cell *c*. Let $X_{C}$ denote the ligand and receptor expression of all cells in the same tissue as cell *c* (including cell *c*). Optionally, let $M_{C}$ denote cell type metadata from those same cells. For each neighborhood radius *r* and each response gene *g*, SPICE tests a hypothesis $H_{r}$ that the conditional expectation of the response gene expression at each cell can be modeled as


$$E[Y_{c,g}|X_{C},M_{C}]=f_{r}(X_{C},M_{C}),$$

where $f_{r}$ refers to our GCN trained on graphs with neighborhood radius *r*. In particular, SPICE tests $H_{r}$ by measuring how closely the corresponding GCN predictions match the observed values. For each gene *g* and each neighborhood radius *r*, we calculate the mean squared difference between the actual observed gene expression, $Y_{c,g}$, and the GCN predicted expression, $f_{r}(X_{C},M_{C})$, within our test dataset. This calculation gives us an unbiased estimate of


$$E\left[{\left|\left|Y_{c,g}-f_{r}(X_{C},M_{C})\right|\right|}^{2}|X_{C},M_{C}\right],$$

which we denote as ${\hat{\sigma }}_{r,g}^{2}$. The exact conditional expectation would minimize this MSE (Hastie *et al*. 2001, Ch. 2). Therefore, we reject the hypothesis $H_{r}$ (that SPICE with radius *r* accurately reflects true expression patterns) if we can find another value $r^{\prime }$ whose estimated MSE ${\hat{\sigma }}_{r^{\prime },g}^{2}$ is substantially smaller than ${\hat{\sigma }}_{r,g}^{2}$. The supplementary material (Section 2) provides additional details about hypothesis testing.

The choice r=0 is of particular interest, as the value of $f_{0}(X_{C},M_{C})$ is invariant to gene expression and metadata outside the target cell *c*. In other words, the hypothesis $H_{0}$ states that a spatially ignorant predictor can accurately model the conditional gene expression. We are particularly interested in the case where ${\hat{\sigma }}_{r,g}^{2}$ is much less than ${\hat{\sigma }}_{0,g}^{2}$ for some *r*, as it suggests that a spatially ignorant model may be inadequate to explain the gene expression, which, in turn, suggests that the expression of gene *g* may exhibit spatial variations.

Other methods, such as MESSI, could also be used to perform such a test. However, we find that SPICE achieves superior predictive accuracy across a variety of settings, suggesting that the MESSI model may not be sufficiently flexible to accurately model the conditional expectations of interest. Thus, adopting MESSI for model comparison could lead to inflated false-negative rates or incorrectly ordered spatial gene rankings.

SIMVI and SpaCeNet can also be used to identify genes exhibiting spatial effects. However, these methods require different inputs and typically require the full gene expression matrix rather than performing supervised prediction of response genes from predefined ligand/receptor predictors. As a result, a direct apples-to-apples comparison with the supervised prediction task considered here is not straightforward (see Discussion).

## 3 Results

We demonstrate SPICE for determining CCC-affected genes in both real and semi-synthetic data. To explore the necessity of highly expressive spatial models, we also compare the predictive power of the SPICE model with prediction schemes that use pre-aggregated representations of cellular neighborhoods, in conjunction with learning algorithms such as LightGBM, mixture of experts (MESSI), ridge regression, and LASSO. For all approaches, the response gene in each target cell is predicted from (i) ligand and receptor expression in the target cell, (ii) average neighborhood ligand and receptor expression, and (iii) average neighborhood metadata (e.g. the relative abundance of different cell types in the neighborhood). Models are evaluated across a range of tissue graph radii to capture mechanisms acting at different spatial scales—from local (paracrine, juxtacrine) to distant (endocrine) signaling. This multi-scale evaluation also helps identify the most relevant neighborhood size for CCC influence and tests model robustness to varying spatial resolutions. All models were thoroughly hyperparameter-tuned to compare the best-performing model within each family; these details are included in Supplementary Section 1.

### 3.1 MERFISH hypothalamus

**Figure 3:**
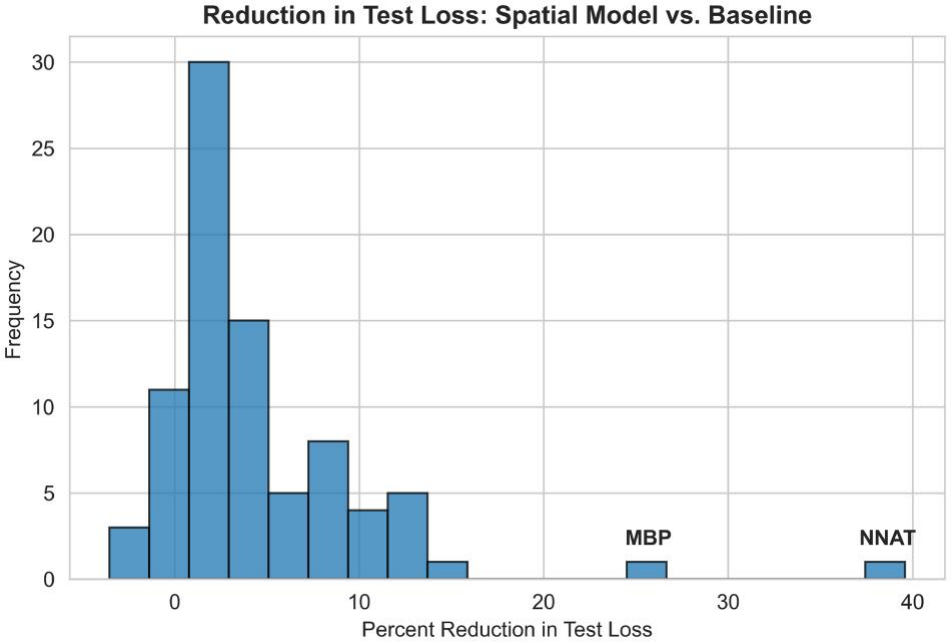
Gene test loss reductions from training the SPICE GCN on tissue graphs with r=25 µm instead of r=0 µm on the MERFISH Hypothalamus dataset.

To ensure generalization, we split the MERFISH hypothalamus data into training, validation, and test sets by animal ID: no animal appears in more than one split. We first evaluate the ability of SPICE to detect spatially regulated genes. For each response gene, we compare SPICE’s predictive performance against that of a baseline estimator. Figure 3 shows the differences. The NNAT and MBP genes stand out, with our GCN achieving 39.59% and 24.81% reductions in MSE, respectively, by considering neighboring signals. MBP has been corroborated to be spatially variable in the brains of mice (Hu *et al*. 2021), and NNAT has been demonstrated to have spatial and temporal patterns during mouse eye development (Sel *et al*. 2017). Across all response genes, 91.6% are better predicted when spatial information is leveraged. Figure 2, available as supplementary data at *Bioinformatics Advances* online provides the MSE improvements for all response genes. The median loss reduction was 2.77%, and the mean was 4.56%.

We next examine predictive model performance on the MERFISH dataset in two settings: with and without cell type metadata. Cell type annotations can help characterize specific behaviors via gene activity but are inconsistent across spatial transcriptomics datasets, varying in boundary criteria and granularity used to define the taxonomy (Lähnemann *et al*., 2020). They may also be a source of data leakage if response genes have been used to infer cell types. We do not take a stance on whether cell type annotations should be included as predictors; instead, we report results for both settings.

**Figure 4:**
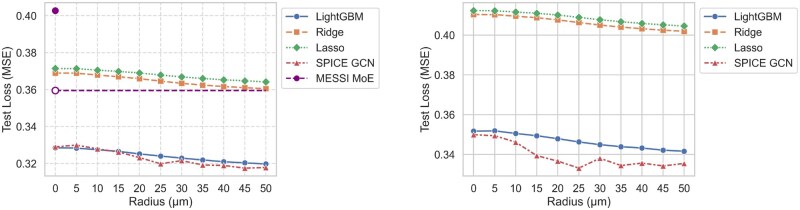
Test MSE on the MERFISH hypothalamus dataset with cell type annotations (left) and without cell type annotations (right). MESSI uses a fixed Delaunay triangulation representation of neighbors; we use a closed purple dot for MESSI’s MSE without neighboring input, and the dashed line represents it with neighboring input.

Figure 4 shows performance on six animals in our test set, evaluated with multiple neighborhood radii. The left panel shows the results when cell type annotations are included as metadata, whereas the right panel shows the results without them. Since MESSI requires filtering by cell type, we report its performance only in the setting where cell types are included. For MESSI, we selected its best-performing cell type: excitatory. Our results indicate that the spatially informed GCN can achieve a 4.79% reduction in loss when cell types are absent and a 3.00% reduction when cell types are included. When cell types are included, LightGBM performs nearly identically to our GCN, which is expected since cell type annotations are strong indicators for CCC influences by capturing shared signaling environments and constraining plausible interaction partners (Cable *et al*. 2022). Methods for detecting CCC interactions typically assume that relevant ligand-receptor pairs are known a priori and enriched within and between specific cell types. When cell-type information is excluded, this assumption becomes a liability: all models have a higher MSE than the spatially ignorant LightGBM or SPICE baseline. Even so, SPICE achieves greater error reductions than the competing methods in this setting.

### 3.2 Xenium fresh frozen mouse brain

We now apply SPICE to the Xenium Mouse Brain dataset. Unlike the previous dataset, this one includes only a single tissue sample, which prevents using separate animals for training and testing. Instead, we spatially partition the data into multiple disconnected subgraphs that can serve as distinct samples for training, validation, and test splits. See the Supplementary Material (Section 1) for a detailed description of the graph partitioning algorithm. Table 1 reports the resulting held-out model performance across a range of neighborhood radii.

**Table 1:** Test MSE for various methods and neighborhood radii on the Xenium Fresh Frozen Mouse Brain dataset.

Model/*r*	0	5	10	15	20	25	30
Ridge	0.245	0.246	0.244	0.237	0.229	0.224	0.221
Lasso	0.511	0.511	0.511	0.511	0.511	0.511	0.511
Elastic Net	0.264	0.264	0.263	0.260	0.256	0.253	0.252
LightGBM	0.187	0.187	0.184	0.180	0.175	0.173	0.172
SPICE GCN	**0.178**	**0.178**	**0.179**	**0.176**	**0.172**	**0.170**	**0.169**

As with our evaluation on the MERFISH dataset, we include several linear models as baseline references, as well as LightGBM, which serves as our primary benchmark due to its competitive performance and scalability. We no longer compare against MESSI because it requires cell type annotations, which are not available in this dataset. Furthermore, we have demonstrated that LightGBM strictly outperforms MESSI on the MERFISH dataset, thereby reducing the need for additional comparisons with the latter. While both LightGBM and SPICE achieve reductions in test error with additional spatial information, SPICE’s non-spatial baseline performance is roughly the same as LightGBM’s performance with a neighborhood radius of 15 µm. These equivalent performances complicate the interpretation of downstream analyses, as the apparent gains from including spatial context become less reliable. A spatial model that performs worse than a non-spatial baseline may be underfitting intrinsic features, leading to incorrect conclusions about spatial dependence. SPICE’s capacity to model nonlinear and hierarchical relationships allows it to make informative predictions based solely on a cell’s intrinsic features. Therefore, when SPICE exhibits improved performance at higher radii, we can be more confident that these gains reflect genuine spatial effects rather than merely compensating for the model’s inability to fully leverage a cell’s intrinsic features. Furthermore, SPICE jointly predicts all genes yet still outperforms per-gene LightGBM models, which suggests that SPICE is more computationally efficient and that joint prediction may provide beneficial regularization.

**Figure 5:**
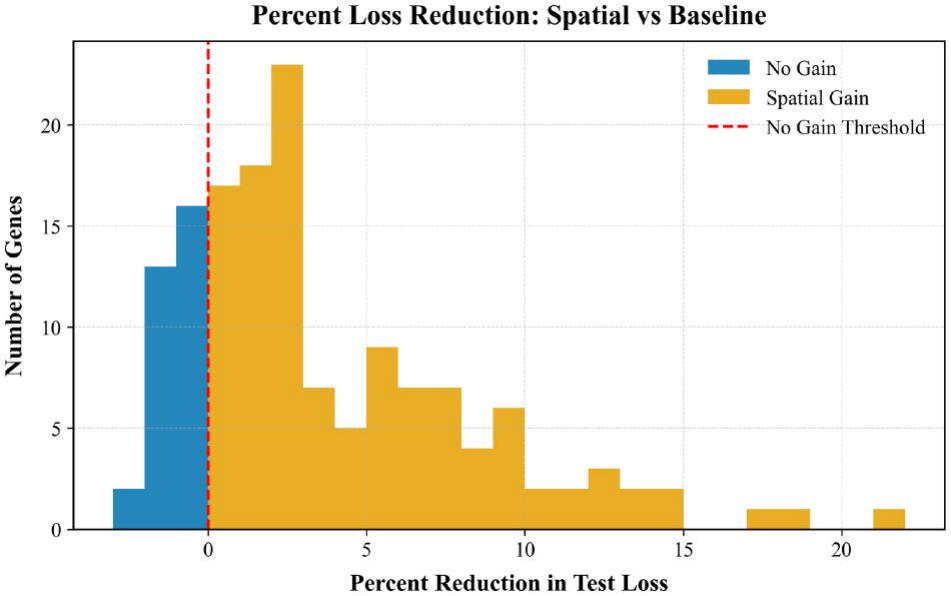
Gene test loss reductions from training our GCN on tissue graphs with r=30 µm instead of r=0 µm on the Xenium Fresh Frozen Mouse Brain dataset.

We can assess which response genes from the Xenium dataset exhibit spatial dependence by comparing a SPICE network with r=30 µm to a spatially ignorant SPICE network (r=0 µm) as a baseline. Figure 3, available as supplementary data at *Bioinformatics Advances* online provides the performance differences for all response genes. Many response genes are somewhat better predicted with a spatial model than the baseline model (see Fig. 5), but a handful of genes show remarkable differences. Established results corroborate the importance of these genes. The CABP7 gene (21.00% MSE reduction) enables calcium ion binding and is highly expressed in subregions of the mouse hippocampus (Shi *et al*. 2023). The GFAP gene (18.16% MSE reduction) is directly affected in Huntington’s disease and appears with spatially distinct expression profiles (Brown *et al*. 2023). Lastly, the ARC gene (17.22% MSE reduction) is primarily concentrated in the CA1 region of the hippocampus, more than in any other brain region (Gao *et al*. 2018). This finding aligns with existing research that suggests ARC upregulation is essential for spatial learning and supports long-term memory storage (Gao *et al*. 2018).

### 3.3 Semi-synthetic experiments

To complement our experiments with real data, for which ground truth is unavailable, we develop experiments on semi-synthetic data to explore how our model can recover response gene expressions in several regimes of potential cell-cell interactions. Our semi-synthetic datasets use cell positions from the MERFISH hypothalamus dataset and simulated gene expression. We start by generating independent and identically distributed random expression values for all genes for each cell. Then, these data are perturbed to reflect a predefined correlation structure between neighboring signal cells and their targets. For each experiment, we predict a single response gene. The true response gene expression is calculated as a function of the selected ligand’s expression in all neighbors of the target cell. For this set of experiments, we assume a true neighborhood radius r=30 µm. Ideally, a model that accurately recovers the response expression would be able to do so when given all of the necessary predictors.

#### 3.3.1 Thresholded-sum signals

In our first semi-synthetic experiment, we generate data by sampling the expression levels for the predictor genes i.i.d. from an exponential distribution. In particular, for cell *c*, the expression level of predictor gene *g* is $X_{c,g}∼\;\text{exp}(10)$. The response gene expression is thresholded by a sum:


$$Y_{c,0}=1\left(S_{c}>1\right)S_{c}+\varepsilon_{c},$$

where $S_{c}=\sum_{c^{\prime }\in N(X_{c})}X_{c^{\prime },1}$ and $\varepsilon_{c}∼\;\text{exp}(10)$.

**Figure 6:**
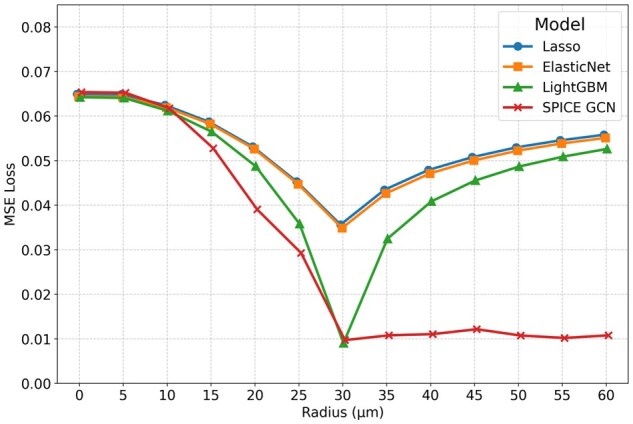
Thresholded sum synthetic experiment results. The true data-generating process has a neighborhood radius of $r^{*}=30$ µm.

This synthetic relationship is intentionally straightforward to test whether models can recover a simple spatial dependence. GCNs and boosting techniques almost perfectly recover the true relationship between neighboring and target expressions. Figure 6 shows that all models attain their best performance at r=30 µm and gain performance advantages on unseen data as *r* increases from 0 µm to 30 µm. However, only SPICE is able to maintain optimal performance when given a broader-than-necessary radius (r>30) µm. Including neighboring information just 5 µm beyond the true neighborhood radius degrades model performance nearly as much as excluding all data between 25 µm and 30 µm for all competing methods. In contrast, the GCN performs adequately over a wide range of neighborhood radii.

#### 3.3.2 Distance-weighted signals

In our second semi-synthetic experiment, we generate data by sampling a hierarchical model in which each predictor gene g=1,…,G has an overall expression level that is sampled independently from a normal distribution: $\mu_{g}∼N(20,4)$. Then, the expression level of predictor gene *g* in cell *c* follows a rescaling of a negative binomial distribution: $X_{c,g}∼\text{NB}(\mu_{g},0.5)/60$.

The expression level of the sole response gene is


$$Y_{c,0}=\sum_{X_{c^{\prime }}\in N(X_{c})}\sqrt{X_{c^{\prime },1}}\left(1-\frac{\sinh^{-1}\left(5.863\;d(X_{c},X_{c^{\prime }})\right)}{5.863}\right),$$

where *d* gives the distance between pairs of cells. This response simulates the influence of ligands from neighboring cells as a nonlinear, inverse function of their distance from the target cell, decaying from 1 to 0 as the neighborhood radius increases from 0 µm to 30 µm. Because the contribution of each neighboring cell is the square root of its ligand expression, the impact of weaker signals is emphasized.

**Figure 7:**
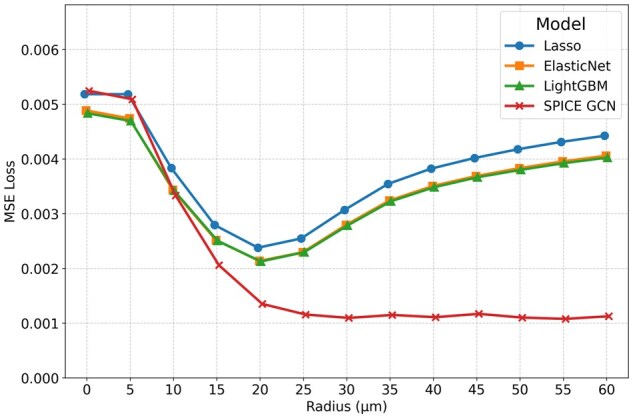
Distance-weighted synthetic experiment results. The true data-generating process has a neighborhood radius of $r^{*}=30$ µm.

Figure 7 demonstrates that in these settings, models with pre-aggregated neighborhood representations not only have higher loss values than our GCN for r>15 µm, but their minimum losses occur at neighborhood radii that differ from the actual interaction radius (20 µm). In contrast, the SPICE prediction error decreases monotonically as the neighborhood radius increases up to 30 µm, then plateaus. This trend indicates that the network was able to ignore irrelevant signals from higher radii. We conjecture that the elevated error observed in models with pre-aggregated neighborhood representations primarily results from their reliance on summarizing neighborhood gene expression by taking the *average* of neighboring expressions. Such averaging fails to capture the spatial relationships in this dataset, where gene expression is driven by a *sum* of neighboring values. Although it would be possible to construct neighborhood representations that better represent this particular spatial dependency if it were known a priori, in real data, these dependencies are seldom known a priori. SPICE captures a wide variety of relationships without prior knowledge of their specific forms, thus eliminating the need for manual design of neighborhood representations.

#### 3.3.3 Commonalities

In both experiments, SPICE’s performance remains stable as the neighborhood radius increases once it reaches a sufficiently large value. In contrast, methods based on pre-aggregated neighborhood representations are highly sensitive to the neighborhood radius. In practice, the true radius of influence is unknown and may vary across cells and genes, exhibit multimodal effects, or depend on complex biological interactions; thus, this stability is desirable. Furthermore, the performance of methods that rely on fixed functional summaries of neighboring expression is non-monotonic as a function of *r*. Non-monotonic performance makes it more difficult to interpret the results from the model-selection perspective given in Section 2.4.

## 4 Discussion

Graph convolutional networks (GCNs) enhance gene expression prediction from spatial transcriptomics data. By comparing spatially informed GCNs to spatially ignorant baselines, we identify CCC-affected genes through reductions in prediction error. This approach is valid when models are sufficiently flexible and accurately estimated. Our empirical results show that SPICE consistently achieves lower mean squared errors against several preceding methods, indicating its effectiveness in capturing genuine cell–cell communication effects. This analysis could be further extended by examining the outputs of SIMVI and SpaCeNet. Both of these methods permit comparisons between spatially informed and spatially ignorant model versions. SIMVI accomplishes this via archetypal analysis and a subsequent spatial effect $R^{2}$ estimation for each gene. SpaCeNet does this by comparing reconstruction errors between models that estimate a precision matrix with and without intercellular model parameters. Direct comparison with these methods is challenging because they differ from SPICE in the input features they use. SIMVI and SpaCeNet use the full gene expression matrix to estimate spatial effects, whereas SPICE estimates response gene expression using curated ligand and receptor predictors, and we follow this precedent set by MESSI. Adding or removing certain inputs changes the model’s underlying inference objective.

The results of any model comparison must be interpreted with caution. Differences in model flexibility and estimation accuracy can affect the validity of the results. A more flexible spatial model might outperform a simpler non-spatial model even when spatial information is irrelevant, or vice versa if the spatial model is poorly estimated. Additionally, measurement noise can create apparent gains from neighborhood information by averaging out random fluctuations rather than capturing true intercellular signaling. Therefore, consideration of model assumptions and noise levels is essential.

Moreover, improvements in predictive accuracy do not necessarily imply causal relationships. Spatial dependencies may arise from confounding factors such as shared developmental lineage or donor-specific effects. To draw meaningful biological conclusions, domain-specific knowledge is required to control for potential confounders. For example, ensuring that training and testing data come from separate tissue donors may help mitigate donor-specific biases.

In summary, while GCNs can be valuable tools for analyzing spatial transcriptomics data, their application requires attention to model flexibility, estimation accuracy, noise considerations, and biological context to yield reliable insights into spatial gene expression patterns.

## Supplementary Material

vbag101_Supplementary_Data

## Data Availability

All data utilized in this work is publicly available. Synthetic dataset generation is included alongside SPICE's code at https://github.com/prob-ml/spice. The MERFISH hypothalamus data can be found at https://datadryad.org/dataset/doi:10.5061/dryad.8t8s248. The Xenium fresh frozen mouse brain sample can be found at https://www.10xgenomics.com/datasets/fresh-frozen-mouse-brain-replicates-1-standard.

## References

[vbag101-B1] 10x Genomics. Fresh frozen mouse brain replicates 1 standard dataset, 2023. https://www.10xgenomics.com/datasets/fresh-frozen-mouse-brain-replicates-1-standard.

[vbag101-B2] Brown TG , Thayer MN, VanTreeck JG et al Striatal spatial heterogeneity, clustering, and white matter association of GFAP+ astrocytes in a mouse model of huntington’s disease. Front Cell Neurosci 2023;17:1094503. 37187609 10.3389/fncel.2023.1094503 PMC10175581

[vbag101-B3] Cable DM , Murray E, Shanmugam V et al Cell type-specific inference of differential expression in spatial transcriptomics. Nat Methods 2022;19:1076–87. 36050488 10.1038/s41592-022-01575-3 PMC10463137

[vbag101-B4] Cang Z , Zhao Y, Almet AA et al Screening cell–cell communication in spatial transcriptomics via collective optimal transport. Nat Methods 2023;20:218–28. 36690742 10.1038/s41592-022-01728-4 PMC9911355

[vbag101-B5] Chami I et al Machine learning on graphs: a model and comprehensive taxonomy. J Mach Learn Res 2022;23:1–64.

[vbag101-B6] Dong M , Su DG, Kluger H et al Simvi disentangles intrinsic and spatial-induced cellular states in spatial omics data. Nat Commun 2025;16:2990. 40148341 10.1038/s41467-025-58089-7 PMC11950362

[vbag101-B7] Fischer DS , Schaar AC, Theis FJ et al Modeling intercellular communication in tissues using spatial graphs of cells. Nat Biotechnol 2023;41:332–6. 36302986 10.1038/s41587-022-01467-z PMC10017508

[vbag101-B8] Fortelny N , Bock C. Knowledge-primed neural networks enable biologically interpretable deep learning on single-cell sequencing data. Genome Biol 2020;21:190. 32746932 10.1186/s13059-020-02100-5 PMC7397672

[vbag101-B9] Foster D , Frost-LaPlante B, Victor C et al Gradient sensing via cell communication. Phys Rev E 2021;103:022405. 33735979 10.1103/PhysRevE.103.022405

[vbag101-B10] Gao X , Castro-Gomez S, Grendel J et al Arc/Arg3.1 mediates a critical period for spatial learning and hippocampal networks. Proc Natl Acad Sci USA 2018;115:12531–6. 30442670 10.1073/pnas.1810125115 PMC6298089

[vbag101-B11] Hastie T et al *The Elements of Statistical Learning. Springer Series in Statistics*. Springer Inc, 2001.

[vbag101-B12] Hou R , Denisenko E, Ong HT et al Predicting cell-to-cell communication networks using NATMI. Nat Commun 2020;11:5011. 33024107 10.1038/s41467-020-18873-z PMC7538930

[vbag101-B13] Hu J , Li X, Coleman K et al SpaGCN: integrating gene expression, spatial location and histology to identify spatial domains and spatially variable genes by graph convolutional network. Nat Methods 2021;18:1342–51. 34711970 10.1038/s41592-021-01255-8

[vbag101-B14] Jin S , Guerrero-Juarez CF, Zhang L et al Inferences and analysis of cell-cell communication using CellChat. Nat Commun 2021;12:1088. 33597522 10.1038/s41467-021-21246-9 PMC7889871

[vbag101-B15] Lähnemann D , Köster J, Szczurek E et al Eleven grand challenges in single-cell data science. Genome Biol 2020;21:31. 32033589 10.1186/s13059-020-1926-6 PMC7007675

[vbag101-B16] Li D , Ding J, Bar-Joseph Z et al Identifying signaling genes in spatial single-cell expression data. Bioinformatics 2021;37:968–75. 32886099 10.1093/bioinformatics/btaa769 PMC8128476

[vbag101-B17] Long Y , Ang KS, Li M et al Spatially informed clustering, integration, and deconvolution of spatial transcriptomics with GraphST. Nat Commun 2023;14:1155. 36859400 10.1038/s41467-023-36796-3 PMC9977836

[vbag101-B18] Mac NA , Nguyen HS. 2021. Rotation invariance in graph convolutional networks. *Ann Comput Sci Inf Syst.*

[vbag101-B19] Marx V. Method of the year: spatially resolved transcriptomics. Nat Methods 2021;18:9–14. 33408395 10.1038/s41592-020-01033-y

[vbag101-B20] Moffitt JR , Bambah-Mukku D, Eichhorn SW et al Molecular, spatial, and functional single-cell profiling of the hypothalamic preoptic region. Science 2018;362. 10.1126/science.aau5324 PMC6482113 30385464

[vbag101-B21] Monti F et al 2017. Geometric deep learning on graphs and manifolds using mixture model CNNs. In *Proceedings of the IEEE Conference on Computer Vision and Pattern Recognition,* 5425–34.

[vbag101-B22] Mortazavi A , Williams BA, McCue K et al Mapping and quantifying mammalian transcriptomes by RNA-seq. Nat Methods 2008;5:621–8. 18516045 10.1038/nmeth.1226 PMC13303166

[vbag101-B23] Satija R , Farrell JA, Gennert D et al Spatial reconstruction of single-cell gene expression data. Nat Biotechnol 2015;33:495–502. 25867923 10.1038/nbt.3192 PMC4430369

[vbag101-B24] Schrod S , Lück N, Lohmayer R et al Spatial cellular networks from omics data with spacenet. Genome Res 2024;34:1371–83. 39231609 10.1101/gr.279125.124 PMC11529864

[vbag101-B25] Sel S , Patzel E, Poggi L et al Temporal and spatial expression pattern of Nnat during mouse eye development. Gene Expr Patterns 2017;23-24:7–12. 28038958 10.1016/j.gep.2016.12.002

[vbag101-B26] Shi H , He Y, Zhou Y et al Spatial atlas of the mouse central nervous system at molecular resolution. Nature 2023;622:552–61. 37758947 10.1038/s41586-023-06569-5 PMC10709140

[vbag101-B27] Strobl C , Boulesteix A-L, Zeileis A et al Bias in random forest variable importance measures: illustrations, sources and a solution. BMC Bioinformatics 2007;8:25–1. 17254353 10.1186/1471-2105-8-25 PMC1796903

[vbag101-B28] Tang F , Barbacioru C, Wang Y et al mRNA-seq whole-transcriptome analysis of a single cell. Nat Methods 2009;6:377–82. 19349980 10.1038/nmeth.1315

[vbag101-B29] Tirosh I , Izar B, Prakadan SM et al Dissecting the multicellular ecosystem of metastatic melanoma by single-cell RNA-seq. Science 2016;352:189–96. 27124452 10.1126/science.aad0501 PMC4944528

[vbag101-B30] Türei D , Valdeolivas A, Gul L et al Integrated intra- and intercellular signaling knowledge for multicellular omics analysis. Mol Syst Biol 2021;17:e9923. 33749993 10.15252/msb.20209923 PMC7983032

[vbag101-B31] Wang Z , Gerstein M, Snyder M et al RNA-seq: a revolutionary tool for transcriptomics. Nat Rev Genet 2009;10:57–63. 19015660 10.1038/nrg2484 PMC2949280

[vbag101-B32] Weber LM , Saha A, Datta A et al nnsvg for the scalable identification of spatially variable genes using nearest-neighbor gaussian processes. Nat Commun 2023;14:4059. 37429865 10.1038/s41467-023-39748-z PMC10333391

[vbag101-B33] Yang Y , Hong Y, Zhao K et al Spatial transcriptomics analysis identifies therapeutic targets in diffuse high-grade gliomas. Front Mol Neurosci 2024;17:1466302. 39530009 10.3389/fnmol.2024.1466302 PMC11552449

[vbag101-B34] Yuan Y , Bar-Joseph Z. GCNG: graph convolutional networks for inferring gene interaction from spatial transcriptomics data. Genome Biol 2020;21:300. 33303016 10.1186/s13059-020-02214-w PMC7726911

