# Supplementary material for: Graph convolutional networks for inferring cell-cell communication from spatial transcriptomics data
Source: Bioinform Adv. 2026 Apr 9;6(1):vbag101. doi: 10.1093/bioadv/vbag101 (PMC13110010; doi:10.1093/bioadv/vbag101)
Supplement: vbag101_Supplementary_Data [file vbag101_supplementary_data.pdf]

Supplementary Material for Graph Convolutional Networks for Inferring Cell-Cell Communication from Spatial Transcriptomics Data

Roman Kouznetsov, Jackson Loper, and Jeffrey Regier

Department of Statistics, University of Michigan

Contents

1 SPICE Additional Model Details **3**

1.1 SPICE Memory, Training and Hardware 3

1.2 Hyperparameter Tuning 4

1.3 Graph Splitting 5

2 Hypothesis testing **8**

2.1 Equivalence of MSE minimization and Gaussian likelihood maximization . . 10

2.2 Connecting likelihood ratio tests to prediction error 10

2.3 Null distribution of test statistic 11

2.4 Gene-level testing 12

2.5 Generalization to unknown variance 12

3 Additional Results **14**

4 An Extended Discussion of the Challenges in Inferring CCC from Spatial Transcriptomics Data **17**

4.1 Flexible models, accurately estimated and appropriately evaluated 17

4.2 Accurate noise models 19

4.3 Connecting model comparison with scientific questions 20

1 SPICE Additional Model Details

1.1 SPICE Memory, Training and Hardware

As the radius of consideration increases, the number of edges increases at $O(r^2)$. The number of values needed to store a forward pass increases at $O(r^2kd)$ where r is the radius of consideration, k is the kernel size, and d is the pseudo-coordinate dimension. Running deeper and wider models may require splitting the model across multiple GPUs. SPICE models leveraged 3 NVIDIA TU102 cards in cases where the input graphs were too large to be stored on a single GPU card. This was accomplished using PyTorch Lightning's Distributed Data Parallel (DDP) strategy, which partitions each batch across devices and synchronizes gradient updates during training (Falcon *et al.*, 2025).

Under the production settings, a single training iteration of the model with a fixed radius averages around 2 minutes and 40 seconds for Xenium data and 21 minutes for MERFISH hypothalamus data. However, these times are variable due to the slightly stochastic behavior of early stopping criteria. Run times will also increase if one wants to ensure that SPICE is trained for a minimum number of epochs before early stopping is permitted.

As Xenium scales up to support thousands of genes, the first layer of SPICE can be prohibitively expensive to train. To scale accordingly would require additional computational resources or some dimensionality reduction of the feature space. Ideally, the dimensionality reduction would preserve gene-level interpretability, allowing us to recover which genes are spatially differentiable.

1.2 Hyperparameter Tuning

SPICE was evaluated for a variety of hyperparameters to identify the best version for generalization. We selected the model with the lowest validation mean squared loss. The winning candidate has the hyperparameters displayed in the tree below. The hyperparameters chosen in production were applied identically for each radius value.

```
├── optimizer
│   └── [Adam, SGD]
│       ├── Learning Rate = [0.001, 0.0001]
│       ├── Weight Decay = 1e-6
│       └── Momentum = 0
├── training
│   ├── Max Training Epochs = [2000, 1200, 1000, 500]
│   └── Early Stopping Patience Epochs = [5, 10, 15]
└── model
    ├── Hidden Layer Width = [128, 256, 512, 1024]
    ├── Number of Hidden Layers = [1, 2, 3, 4, 5, 6]
    ├── # of GMM Kernels = [2, 5, 10, 25]
    ├── Skip Connections = [True, False]
    ├── Batchnorm = [True, False]
    ├── Dropout = [0, 0.05, 0.1, 0.2]
    ├── Edge Attributes = [Euclidean Distance, Polar Coordinates, Node Degree,
    │   Polar Coordinates & Network Degree]
    └── Aggregation Method = ["mean", "sum"]
```

To ensure a fair comparison against LightGBM, MESSI, and linear models, we tuned plausible hyperparameters for each model family and evaluated their best-performing versions.

Using LightGBM's gradient boosting type for the regression task evaluated with MSE, the hyperparameters we considered are listed in the tree below. Elements underlined in blue indicate the final hyperparameter chosen for test set evaluation.

```
└── tuning grid
    └── Learning Rate = [0.1, 0.01, 0.005, 0.001]
```

```
├── n_estimators = [100, 300, 500]
├── max_depth = [3, 5, 7]
└── min_data_in_leaf = [15, 20, 30]
```

Similarly, the penalty values considered for Ridge and Lasso are outlined below. Lasso's best value was $\alpha = 0.01$ while Ridge's optimal value was $\alpha = 5.0$.

```
└── tuning grid
    └── α = [0.01, 0.05, 0.1, 0.25, 0.5, 1.0, 2.5, 5.0]
```

Finally, the penalty values applied to ElasticNet are:

```
└── tuning grid
    ├── α = [0.01, 0.05, 0.1, 0.25, 0.5, 1.0, 2.5, 5.0]
    └── L1 Ratio = [0.01, 0.05, 0.1, 0.25, 0.5, 0.75, 0.95, 0.99]
```

MESSI performed a grid search of their own, and their published source code comes pre-configured with the best-performing hyperparameters for each dataset based on validation error. Each cell type is associated with a specific number of experts, and a weighting scheme (soft or hard) over them. We followed the authors' recommended settings.

1.3 Graph Splitting

In the main text, we evaluated SPICE on the MERFISH hypothalamus dataset and the Xenium Fresh Frozen Mouse Brain dataset. In the latter, we emphasized that only a single tissue was present in the dataset and that in order to evaluate SPICE, we split the dataset into disconnected subgraphs. These subgraphs were then placed into either a train or test set. This section describes the procedure used to split the input tissue graph into smaller, non-overlapping subgraphs for evaluating SPICE on the Xenium dataset.

The limitation of including additional neighbors for each node is the memory needed to store the entire graph. If cell positions are distributed uniformly at random across the tissue slide, then the number of neighbors increases at a rate of $\mathcal{O}(r^d)$, where r is the radius

of consideration and d is the number of spatial dimensions. To reduce the memory of each graph, we can divide a graph into smaller but representative subgraphs (Figure S1).

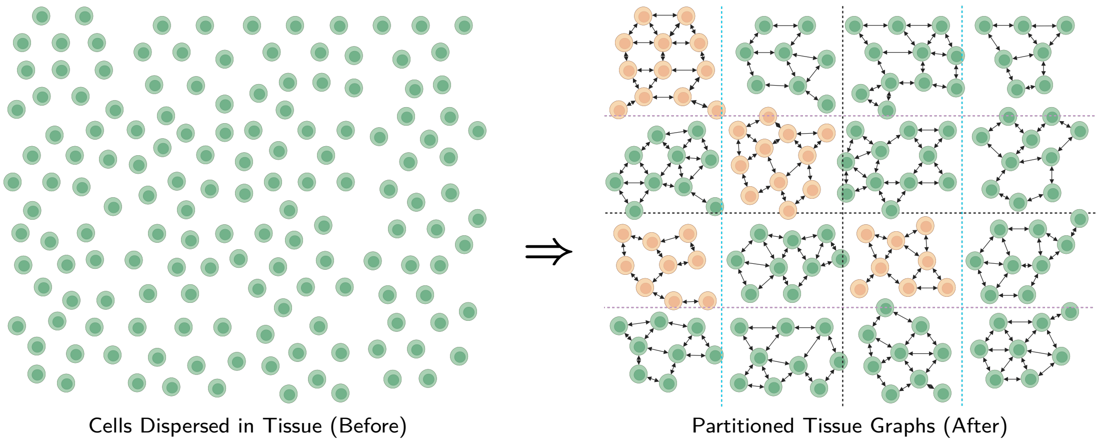

Figure S1: Spatial graph construction and partitioning. In this example, tissue is divided into 16 non-overlapping spatial subgraphs by recursively splitting the tissue spatially at the midpoint (median coordinate) of each axis. Each subgraph is an observation in the batched dataset. These disjoint graphs are split into a training and validation sets (both in green) and a testing set (orange).

The graph splitting algorithm takes as input the graph generated from the original tissue source. We refer to this as the source graph. The splitting procedure then outputs 4 graphs, each containing 25% of the cells from the original graph, as outlined in Algorithm 1.

In the case where there is limited input data, this would be a way to create more training examples for the model to use, though at the cost of a higher dependence between samples generated from the same tissue. Furthermore, the graph splitting ensures model generalization in cases where the data includes only a single tissue, such as the Xenium Fresh Frozen Mouse Brain data.

Algorithm 1 Graph Splitting (2D Case)

Require: $N \geq 0$ ▷ N: The number of rounds of splits
Require: Graph $\mathcal{G}$ ▷ The source graph
Require: Each node in graph $\mathcal{G}$ has spatial coordinates (x, y)
Ensure: List $\mathcal{Q}$ ▷ The split graphs to be used in models

$\mathcal{Q} \leftarrow [\mathcal{G}]$ ▷ $\mathcal{Q}$: Queue of graphs to be split
$\mathcal{Q}_{\text{new}} \leftarrow []$
while $N \neq 0$ **do**
  for all $g \in \mathcal{Q}$ **do**
    $X_0 \leftarrow g((x, y) : x < \text{MEDIAN}(g(x)))$
    $X_1 \leftarrow g((x, y) : x \geq \text{MEDIAN}(g(x)))$
    $X_{00} \leftarrow X_0((x, y) : y < \text{MEDIAN}(X_0(y)))$
    $X_{01} \leftarrow X_0((x, y) : y \geq \text{MEDIAN}(X_0(y)))$
    $X_{10} \leftarrow X_1((x, y) : y < \text{MEDIAN}(X_1(y)))$
    $X_{11} \leftarrow X_1((x, y) : y \geq \text{MEDIAN}(X_1(y)))$
    $\mathcal{Q}_{\text{new}} \leftarrow \mathcal{Q}_{\text{new}} \cup \{X_{00}, X_{10}, X_{01}, X_{11}\}$
  end for
  $\mathcal{Q} \leftarrow \mathcal{Q}_{\text{new}}$
  $\mathcal{Q}_{\text{new}} \leftarrow []$
  $N \leftarrow N - 1$
end while
return $\mathcal{Q}$ ▷ Final split graphs

While each graph has fewer communication events considered, the split is done so that the diameter of the split graph does not fall beneath realistic communication distances. Performing a grid search under various radii of consideration, it became clear that utilizing graphs with radii $>\approx 60\ \mu$m leads to overfitting. Random segmentation is another strategy that has merit because it avoids crafting overly regular graphs; however, this approach risks having more cells near boundary points, severing more of the relevant edges that could facilitate CCCs. This recursive median-based splitting strategy preserves local spatial structure and cell density within each subgraph while minimizing systematic boundary effects across successive splits.

2 Hypothesis testing

In the main text, we said that SPICE tests a hypothesis H_r that the conditional expectation of the response gene expression at each cell can be modeled as

$$\mathbb{E}[Y_{c,g} \mid X_C, M_C] = f_r(X_C, M_C),$$

where f_r refers to our GCN trained on graphs with neighborhood radius r. Furthermore, we emphasized the setting of comparing H_0 to H_{r*} where $r^* > 0$ µm, since that would test if a spatially ignorant model may be inadequate to explain the gene expression.

Ultimately, our goal is to identify spatially dependent genes, so we require an appropriate decision rule to reach and support such conclusions. Evaluating SPICE's performance through MSE reduction provides direct insight into spatial gene dependence. Minimizing the MSE of predictions generated by SPICE is equivalent to maximizing a fixed scale Gaussian distribution. Therefore, we can design a hypothesis test as a principled approach to identify genes with significant spatial effects and prioritize them for further biological validation.

We demonstrate in the coming sections that SPICE is a more appropriate model for spatial dependence inference. Proceeding from this, we can use SPICE predictions in the following hypothesis test:

$$H_0 : \mu_{c,g} = f_{r=0}(X_{C,L}, X_{C,R}, M_C) \text{ v. } H_1 : \mu_{c,g} = f_{r=r^*}(X_{C,L}, X_{C,R}, M_C).$$

As shorthand, we define $\mu_0 := f_{r=0}(X_{C,L}, X_{C,R}, M_C)$ and $\mu_{r^*} := f_{r=r^*}(X_{C,L}, X_{C,R}, M_C)$.

Because the models already provide predictions and we assume fixed variance, we can compute the likelihood ratio directly:

$$\Lambda(Y_{c,g}) = \frac{\mathcal{L}(\mu_0 \mid Y_{c,g})}{\mathcal{L}(\mu_{r*} \mid Y_{c,g})}.$$

Therefore, a likelihood ratio test (LRT) can be performed between a spatially ignorant model and a spatially aware one. We prove that this LRT is proportional to the difference in squared errors under each model, given by:

$$(Y_{c,g} - \mu_{r^*})^2 - (Y_{c,g} - \mu_0)^2.$$

Furthermore, if we use the decision rule $\Lambda < c$ to reject the null hypothesis such that $P(\Lambda < c \mid H_0) = \alpha$ and fail to reject otherwise, then—by the Neyman-Pearson lemma—this is the most powerful α-level test for detecting whether incorporating spatial information improves prediction accuracy under the Gaussian likelihood assumption (Neyman and Pearson, 1933). The proof of this claim as well as derivations of the test statistic, its distribution, and satisfaction of the Neyman-Pearson lemma assumptions can be found in the subsections to follow.

Using $\mathcal{L}(\mu, \sigma)$ to denote the Normal likelihood with mean μ and scale σ, we can create the following likelihood ratio statistic leveraging our predictions:

$$\Lambda = \frac{\mathcal{L}\left(f_{r=0}(X_{C,L}, X_{C,R}, M_C), \sigma\right)}{\mathcal{L}\left(f_{r=r^*}(X_{C,L}, X_{C,R}, M_C), \sigma\right)}.$$

Methods preceding ours could also be used to perform such a test, but we show that their handling of spatial information is often less effective, potentially leading to inflated false positive rates or incorrectly ordered spatial gene rankings. In contrast, SPICE leverages GCNs to better capture spatial dependencies, resulting in improved predictive accuracy and a more reliable evaluation of spatial effects. Providing a statistically rigorous way to rank genes helps determine genes to prioritize when allocating assets for gene research.

2.1 Equivalence of MSE minimization and Gaussian likelihood maximization

SPICE is trained by minimizing the mean squared error, defined as

$$\min_{\hat{Y}} \frac{1}{CG} \sum_{c=1}^{C} \sum_{g=1}^{G} (Y_{c,g} - \hat{Y}_{c,g})^2.$$

If each $Y_{c,g}$ follows a Gaussian distribution with fixed variance and that SPICE provides the predicted mean $\hat{Y}_{c,g}$, then

$$\begin{aligned}
\max_{\hat{Y}} p(\boldsymbol{Y} \mid \hat{\boldsymbol{Y}}) &= \max_{\hat{Y}} \prod_{c=1}^{C} \prod_{g=1}^{G} \frac{1}{\sqrt{2\pi}\sigma} \exp\left\{ -\frac{(Y_{c,g} - \hat{Y}_{c,g})^2}{2\sigma^2} \right\} \\
&= \max_{\hat{Y}} \sum_{c=1}^{C} \sum_{g=1}^{G} \left[\log\left(\frac{1}{\sqrt{2\pi}\sigma} \right) - \frac{(Y_{c,g} - \hat{Y}_{c,g})^2}{2\sigma^2} \right] \\
&= \max_{\hat{Y}} \sum_{c=1}^{C} \sum_{g=1}^{G} - \frac{(Y_{c,g} - \hat{Y}_{c,g})^2}{2\sigma^2} \\
&= \max_{\hat{Y}} \frac{1}{CG} \sum_{c=1}^{C} \sum_{g=1}^{G} -(Y_{c,g} - \hat{Y}_{c,g})^2 \\
&= \min_{\hat{Y}} \frac{1}{CG} \sum_{c=1}^{C} \sum_{g=1}^{G} (Y_{c,g} - \hat{Y}_{c,g})^2.
\end{aligned}$$

2.2 Connecting likelihood ratio tests to prediction error

The test statistic $\Lambda(Y_{c,g})$ can be simplified to a ratio of exponentials:

$$\begin{aligned}
\Lambda_{c,g} &= \frac{(\sqrt{2\pi}\sigma_g)^{-1} \exp\left(-(2\sigma_g^2)^{-1} (Y_{c,g} - \mu_0)^2 \right)}{(\sqrt{2\pi}\sigma_g)^{-1} \exp\left(-(2\sigma_g^2)^{-1} (Y_{c,g} - \mu_{r^*})^2 \right)} \\
&= \frac{\exp\left(-(2\sigma_g^2)^{-1} (Y_{c,g} - \mu_0)^2 \right)}{\exp\left(-(2\sigma_g^2)^{-1} (Y_{c,g} - \mu_{r^*})^2 \right)}.
\end{aligned}$$

If we switch to a decision rule in the log space, we still have the decision rule $\log(\Lambda) < \log(c) = c^*$. We can express the log likelihood ratio test as follows:

$$\begin{aligned}
\log(\Lambda_{c,g}) &= -\frac{1}{2\sigma_g^2}(Y_{c,g} - \mu_0)^2 - \left(-\frac{1}{2\sigma_g^2}(Y_{c,g} - \mu_{r^*})^2\right) \\
&= \frac{1}{2\sigma_g^2}((Y_{c,g} - \mu_{r^*})^2 - (Y_{c,g} - \mu_0)^2) \\
&\propto (Y_{c,g} - \mu_{r^*})^2 - (Y_{c,g} - \mu_0)^2 \\
&= (Y_{c,g}^2 - 2Y_{c,g}\mu_{r^*} + \mu_{r^*}^2 - Y_{c,g}^2 + 2Y_{c,g}\mu_0 - \mu_0^2) \\
&= (2Y_{c,g}(\mu_0 - \mu_{r^*}) + \mu_{r^*}^2 - \mu_0^2).
\end{aligned}$$

Therefore, we have a likelihood ratio test statistic that can be the difference between spatially aware and spatially ignorant model performances.

2.3 Null distribution of test statistic

The test statistic derived from the difference in test MSEs between spatially aware and spatially ignorant models emits a tractable distribution. If H_0 is true, then $\mu_{c,g} = \mu_0$ and $Y_{c,g} \sim N(\mu_0, \sigma_g^2)$. Because the log likelihood ratio test statistic is a composition of affine transformations being applied to $Y_{c,g}$, we can show that it too has a normal distribution:

$$\begin{aligned}
Y_{c,g} &\sim N(\mu_0, \sigma_g^2) \\
2Y_{c,g} &\sim N(2\mu_0, 4\sigma_g^2) \\
2Y_{c,g}(\mu_0 - \mu_{r^*}) &\sim N\Big(2\mu_0(\mu_0 - \mu_{r^*}), 4\sigma_g^2(\mu_0 - \mu_{r^*})^2\Big) \\
2Y_{c,g}(\mu_0 - \mu_{r^*}) + \mu_{r^*}^2 - \mu_0^2 &\sim N\Big(2\mu_0(\mu_0 - \mu_{r^*}) + \mu_{r^*}^2 - \mu_0^2, 4\sigma_g^2(\mu_0 - \mu_{r^*})^2\Big).
\end{aligned}$$

Since our test compares two simple hypotheses with fixed means under a shared, known

variance, and our test statistic admits a closed-form distribution with a decision rule $\Lambda_{c,g} < c$, the assumptions of the Neyman-Pearson lemma are satisfied. Therefore, we have the most powerful α-level test for detecting spatial dependence in gene expression under the fixed-variance Gaussian likelihood model.

2.4 Gene-level testing

To assess spatial dependence at the gene level, we consider the average of the likelihood ratio test statistics across cells. Since we have shown that each $\log \Lambda_{c,g}$ is Gaussian, the average test statistic over all cells C can be written as

$$\bar{T}_g = \frac{1}{C} \sum_{c=1}^{C} \log \Lambda_{c,g}$$

with

$$\bar{T}_g \sim N\left(2\mu_0 \left(\mu_0 - \mu_{r^*}\right) + \mu_{r^*}^2 - \mu_0^2, \frac{4\sigma_g^2 \left(\mu_0 - \mu_{r^*}\right)^2}{C}\right).$$

Under the null hypothesis, we assume each $\mu_{c,g}$ is accurately predicted by a spatially ignorant SPICE. This provides a valid basis for global testing of spatial dependence at the gene level.

2.5 Generalization to unknown variance

We have demonstrated the role of the MSE in testing differences between two means for a Gaussian distribution with a fixed scale. However, the fixed scale assumption may be erroneous. Even in this case, we can use the MLE estimate for the variance to show that the difference in testing mean square errors of model pairs computes the test statistic. Because the MLE estimate for the variance of a Gaussian distribution is exactly the training mean squared error, if we define l_0 and l_{r^*} as the training MSE of SPICE with $r = 0$ and $r = r^*$,

respectively, we can craft an alternate LRT:

$$\Lambda = \frac{(\sqrt{2\pi l_0})^{-1} \exp\left(-(2l_0)^{-1}(Y_{c,g} - \mu_0)^2\right)}{(\sqrt{2\pi l_{r^*}})^{-1} \exp\left(-(2l_{r^*})^{-1}(Y_{c,g} - \mu_{r^*})^2\right)}.$$

Moving to log space, we obtain

$$\begin{aligned}\log(\Lambda) &= -\frac{1}{2}\log l_0 - \frac{1}{2l_0}(Y_{c,g} - \mu_0)^2 - \left(-\frac{1}{2}\log l_{r^*} - \frac{1}{2l_{r^*}}(Y_{c,g} - \mu_{r^*})^2\right) \\ &= \frac{1}{2}(\log l_{r^*} - \log l_0) + \frac{1}{2}\left(\frac{1}{l_{r^*}}(Y_{c,g} - \mu_{r^*})^2 - \frac{1}{l_0}(Y_{c,g} - \mu_0)^2\right).\end{aligned}$$

Unfortunately, this test statistic does not emit a simple or tractable distribution, but a permutation test can be used to construct an empirical null distribution for hypothesis testing. Still, conclusions about spatial dependence assume accurate values for the test MSE values, so SPICE proves to be a reliable framework for this task, offering improved robustness in spatial dependence inference.

3 Additional Results

In the main text, we reported the relative improvements in model performance for the top candidate response genes exhibiting spatial dependence in both real datasets. Figure S2 extends these results to all response genes in the MERFISH hypothalamus dataset and Figure S3 does so for the Xenium dataset.

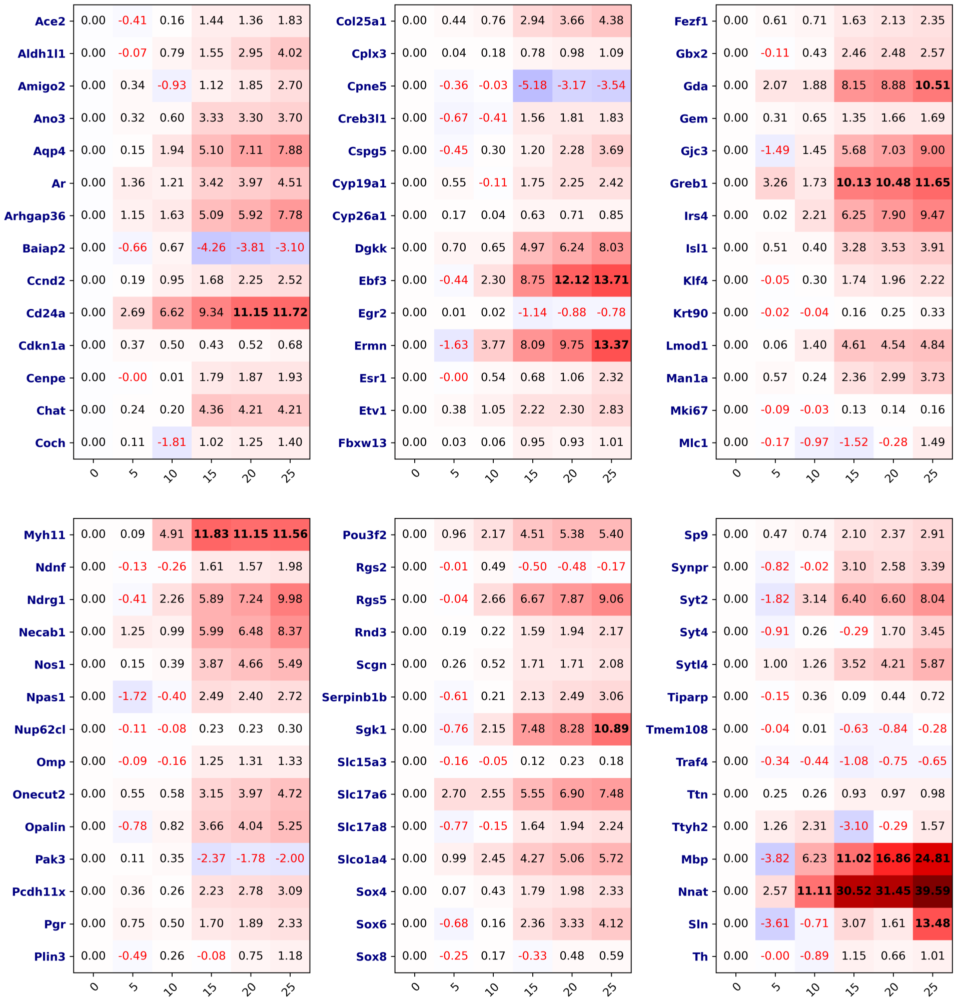

Figure S2: Relative improvements in gene prediction accuracy from SPICE trained on graphs with neighborhood radii from $r = 0\,\mu$m to $r = 25\,\mu$m for the MERFISH dataset. The heat map highlights genes that benefit from spatial information, with warmer colors indicating greater improvement. The black and red numbers indicate the percent reduction in MSE relative to the model trained without spatial information ($r = 0\,\mu$m).

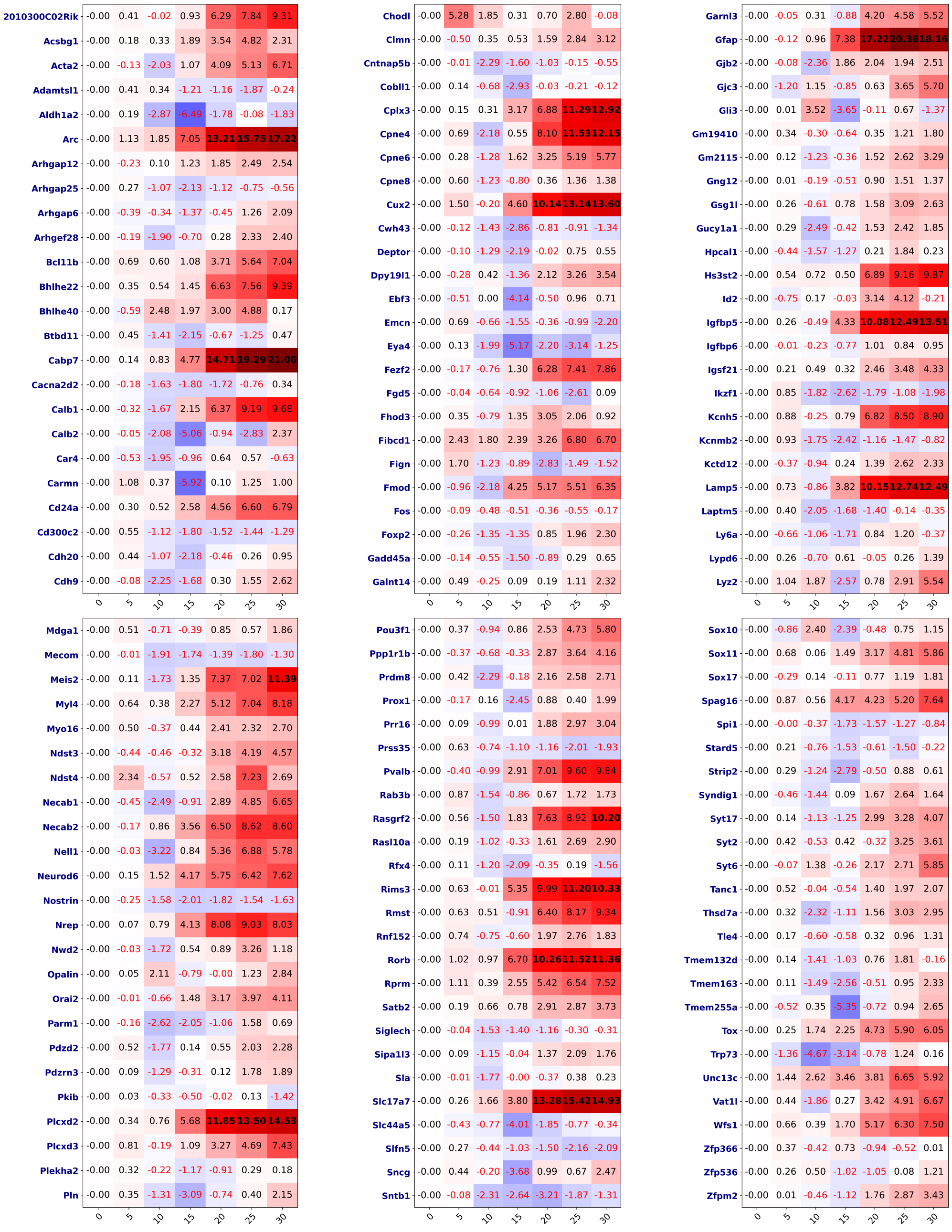

Figure S3: Relative improvements in gene prediction accuracy from SPICE trained on graphs with neighborhood radii from $r = 0\,\mu$m to $r = 30\,\mu$m for the Xenium dataset. The heat map highlights genes that benefit from spatial information, with warmer colors indicating greater improvement. The black and red numbers indicate the percent reduction in MSE relative to the model trained without spatial information ($r = 0\,\mu$m).

4 An Extended Discussion of the Challenges in Inferring CCC from Spatial Transcriptomics Data

In the discussion of the main text, we concluded with the assessment that GCNs serve as better regression models for spatial transcriptomics data and how the SPICE framework is effective in capturing cell-cell communication effects. However, we also acknowledged that interpreting performance differences between spatially ignorant and spatially aware versions of models (even for SPICE) requires great care. This supplement expands on these concerns.

4.1 Flexible models, accurately estimated and appropriately evaluated

Our model comparison framework assumes that each candidate model, given its specific inputs, produces the predictions that minimize average error. This assumption is only viable if the models are all sufficiently flexible and accurately estimated. To see how this could create difficulties, we first consider two abstract examples. In both cases, assume that the spatially ignorant model class is less flexible and the spatially informed model class is more flexible. First, if the target gene depends solely on local (non-spatial) node attributes, a spatially informed model might still outperform a simpler spatially ignorant model. This could occur because the greater flexibility of the spatially informed model allows it to capture complex relationships with local attributes, even though spatial information isn't relevant. Second, if the target gene depends on neighborhood gene expression, a spatially ignorant model might still outperform a spatially informed model. This can happen if estimating the spatially informed model's many parameters requires more data than available, making it difficult to estimate accurately. Consequently, the simpler spatially ignorant model may perform better on held-out data.

In practice, choices about flexibility, estimation, and error can be challenging to reason about. Even minor subtleties can have outsized impacts. Seemingly straightforward model comparisons can become misleading when subtle interactions between flexibility, estimation accuracy, and evaluation criteria are overlooked. Careful attention to these interactions is essential to reliably identify spatially varying genes.

For example, consider the comparisons between a mixture of experts models and gradient boosting models, as performed by Li *et al.* (2021). In these comparisons, the models were evaluated based on the *absolute* difference between response gene expression and predicted gene expression. However, the gradient boosting models were trained to minimize the *squared* difference. It is difficult to interpret comparisons of absolute error in models trained to minimize squared error. In reproducing the results of Li *et al.* (2021), we encountered some of the consequences of this difficulty directly. Although the mixture of experts model yielded smaller absolute errors than a gradient boosting model trained to minimize squared error, as suggested in the original article, we found that the mixture of experts model was dominated by a gradient boosting model trained to minimize absolute error.

For another example, consider the comparisons performed by Fischer *et al.* (2023) with an autoencoder model. The autoencoder model included two parts: an encoder that represented a cell's gene expression in a low-dimensional latent space, and a decoder that predicted gene expression from a position in this latent space. In the reconstruction tasks from the original article, the autoencoder was used to predict local gene expression from neighborhood information as follows: neighborhood information and the *true gene expression* were both given to the encoder to obtain a position in the latent space, and the latent position was then used to predict the gene expression. Interpreting the accuracy of such predictions is challenging because the model predicts a value that was already

provided to it as input. As Fischer *et al.* (2023) notes, the autoencoder model "did not consistently capture spatial dependencies because niche states were represented in latent variables."

For a final example, consider our findings in Table 1 of the main text. The rows of this table for the LightGBM and SPICE models show the broad trend we might expect: averaging over all response genes (including some genes with spatial dependence), spatially informed models outperform spatially ignorant models. However, comparing the two rows leads to a puzzling observation. The spatially ignorant SPICE model obtained a MSE of 0.178, whereas the spatially informed LightGBM model with neighborhood radius $r = 15$ obtains a higher MSE of 0.180. This comparison, taken in isolation at face value, appears to run counter to our knowledge that at least some of the response genes depend on spatial covariates. In view of the rest of the table, we see the comparison's true meaning: even the spatially informed versions of LightGBM are inadequately flexible and/or inadequately estimated.

4.2 Accurate noise models

Consider a scenario in which the true expression of a response gene in a given cell is entirely determined by the expression of gene X in that same cell. Thus, the response gene has no spatial dependence once we condition on the expression of gene X. Assume further that the expression of gene Z in neighboring cells is somewhat predictive of the expression of gene X. Despite the dependency between gene Z and gene X, if we use flexible models, accurately estimated, with appropriate evaluations, we should then find that spatially informed predictions of the response gene are no better than spatially ignorant ones.

The logic above only holds if all measurements of gene expression are noiseless. In practice, this is unrealistic: we do not have exact knowledge of each gene's expression

in each cell. Instead, we have noisy measurements. If these measurements are sufficiently noisy, a spatially informed model (using noisy measurements of the local expression of gene X and noisy measurements of the neighborhood expression of gene Y) may outperform a spatially ignorant model (using only noisy measurements of the local expression of gene X). In this case, the improvement in performance is not due to a spatially varying response gene. Instead, it is simply due to the spatial model's ability to denoise the signal by leveraging correlated measurements across neighboring cells.

In this article, we have assumed that the level of the noise is sufficiently small that this effect can be disregarded. However, a more principled approach could be obtained by identifying a plausible upper bound on the measurement noise. Based on this upper bound, it would be possible to determine if the difference in performance between spatially ignorant and spatially informed models could be due to noise concerns. This could be a fruitful area of exploration for future work.

4.3 Connecting model comparison with scientific questions

Model comparison assesses how altering the information available to the model changes its predictive accuracy. Such assessment is almost never intrinsically interesting, but valuable rather as a lens for answering questions of scientific interest. We conclude this discussion by considering some of the challenges that arise as we attempt to use this lens to answer questions of scientific interest.

For example, consider a question of the form: "is this response gene's expression influenced by cell-cell communication?" To address this question, let us assume we take a model comparison approach and find that the response gene's expression does appear to depend on the gene expression of neighboring cells. This finding may arise from at least two possible causes. First, the response gene may be responding to cell-cell communications.

If this cause is responsible for the observed dependency, then we have arrived at an answer to our question. However, the dependency may arise from a different cause altogether. In every cell, every gene's expression is determined in part by epigenetic states inherited from the cell's ancestors. If the cell's ancestors inhabited the same region of tissue as the cell itself, correlations in gene expression across neighboring cells could reflect complex developmental processes rather than active signaling. Disentangling current signaling from developmental patterns requires domain-specific knowledge of the relevant genes and tissues. More generally, using differences in predictive power to support specific mechanistic claims requires careful integration of the biological context.

For another example, consider a question of the form: "is the expression of this gene in this tissue donor dependent on the expression of genes in neighboring cells?" This question, at first glance, appears to be addressed quite directly by a naiïve model comparison. However, the insertion of the phrase "in this tissue donor" introduces a variety of complications. Consider a scenario in which (i) for each tissue donor the response gene is independent of neighborhood gene expression, (ii) the response gene varies depending on traits of the individual tissue donor, and (iii) neighborhood gene expression can be used to identify those individual traits. In this case, a spatially informed model could outperform a spatially ignorant model, simply because the spatially informed model can effectively identify traits of the tissue donor and use those to better predict the response gene. This identification may be particularly trivial if the model is trained on tissues from one individual and tested on separate tissues from the same individual. Meaningful scientific inquiry thus requires us to track donor effects carefully and attend to the way we split our data between training and testing. For example, a clearer answer to the scientific question in this example might be obtained by allowing all models access to a low-dimensional summary of each tissue donor and ensuring that the training data and testing data come from separate tissue donors.

In brief, the lens of model comparison, like all lenses, has both clarifying power and inherent distortion. When used thoughtfully, model comparison can highlight functional dependencies and suggest mechanisms worth further exploration. However, it can be misleading without rigorous attention to issues such as model flexibility, estimation quality, error quantification, noise, and biological context. Our work suggests that machine learning tools such as GCNs can be helpful in addressing some of these subtle issues.

References

Falcon, W. *et al.* (2025). Pytorchlightning/pytorch-lightning: 2.5.1 release.

Fischer, D. S. *et al.* (2023). Modeling intercellular communication in tissues using spatial graphs of cells. *Nature Biotechnology*, **41**(3), 332–336.

Li, D. *et al.* (2021). Identifying signaling genes in spatial single-cell expression data. *Bioinformatics*, **37**(7), 968–975.

Neyman, J. and Pearson, E. S. (1933). On the problem of the most efficient tests of statistical hypotheses. *Philosophical Transactions of the Royal Society of London. Series A, Containing Papers of a Mathematical or Physical Character*, **231**, 289–337.
